# Case of Cephalic Version by External Manipulation

**Published:** 1879-05-20

**Authors:** 


### *CASE OF CEPHALIC VERSION BY EXTERNAL MANIPULATION.*

Dr. Chesney, in Obstetric Gazette, says:

On the first of February, past, I was requested by Mr. M. to "be at home" about the middle of March, as his wife expected to be confined about that time. I had been the physician of the family for the past five years, and during that time his wife had had two confinements; but the family had been careful to employ only physicians of large experience and eminent ability, as they thought, on these occasions, the lady having on previous occasions suffered from alarming post partum hemorrhage.

My friend, on the day before mentioned, told me that Dr. C., of this city, a gentleman of leading ability in the profession, had attended his wife at her last confinement, and that he had given her something to prevent the flooding; and that he would bring the bottle and let me see what the good Dr. so sagaciously bethought him to use, so that I might fortify myself with the elixir vitæ when he came for me. I promised my good friend that I would be "on hand" at the required moment, but modestly suggested to him that I was not in the habit of consulting the labels on bottles dispensed by other physicians for information as to the remedies I used; and, moreover, I considered myself the equal in all respects, and more particularly so in obstetrics, to the worthy gentleman heretofore employed; and that while I could make no promises as to the result, I would do the best I could for them. He said she had never had any trouble save from hemorrhage. I knew her to have that diathesis strongly marked, as I had been once called to her in the middle of the night to arrest hemorrhage occasioned by the extraction of a tooth, and had attended her oldest little boy also for a most dangerous epistaxis; indeed the whole family are the victims of this dangerous constitutional tendency. But now to my story:

I was called at the expected time, and as it was some miles in the country, I carried with me all I supposed the case would require. She had had pain for some hours, but examination showed that no progress had been made. The os uteri was patulous enough to readily admit the finger, but above it there was nothing in reach. This discovery was significant. By external inquiry I found the child's head lying on the left iliac fossa and the breech to the right. I prognosticated a mean malposition, and trouble, when labor did occur. The pains ceased, and, from the condition of the cervix and os, I concluded labor might, perhaps, be delayed some days; and after a stay of some hours I returned home enjoining upon the family the necessity of communicating the fact to me immediately upon the return of the pains. I was recalled at nightfall.

The case had progressed seemingly very little, although she had had slight pains most all day. The os was, however, very soft and yielding, and I could feel, lying above it, and very movable, what I took to be the left hand. External examination of the venter of the mother showed the position of the child to be the same as in the morning. I began now to wish that I had in this instance, also, been displaced by one of my more illustrious medical brethern, who had so successfully officiated on former occasions; but being *in*, I made up my mind to get out manfully. I had ample time and could have called a consultation. But honest contemplation didn't reveal to me where another physician could profit either me or the patient, and I, therefore, courageously determined to meet the emergency on my own responsibility. I kept all to myself, but at the same time the family and friends were nervous and inquisitive, and, indeed, gave me much more trouble than did the apprehensions in regard to the conditions of the patient. The lady was, however, a person of good sense and courage, which made some amends.

The teachings as to what ought to be done, were plain. "Turn and deliver,"—a thing much easier and safer said than done in any case. I have a horror of a hand in a womb, and make that the dernier resort in all cases. Dr. Robert Lee well says: "I consider it impossible in any case to turn without more or less injury to the mother;" and this has always been a maxim with me. I therefore determined to correct the abnormal positions by external manipulations, and succeeded in doing so in this way:

With my right hand I firmly grasped the head and forcibly pushed it into the uterine cervix, while with the left I moved the breech and lower extremities toward the fundus—a maneuver prosecuted without the least trouble. I now directed an assistant to relieve my left hand, holding the breech firmly in place. I then used my left hand to hold the head in the cervix, when with the right, I made a vaginal inquiry and found the head presenting at the os, while the tip of one hand could be felt below it. Remembering the great tendency in such cases to resume their former positions–to prevent this and secure the advantage already gained—I ruptured the membranes, gave ergot freely, and, in the course of an half hour or so, she had pains and contractions of sufficient force to make the position safe. She had no pain during my manipulations. When pain occurred the hand retreated, and the head was forced well into the superior strait. As soon as the ergotism ceased the pain ceased; and, as the labor was all yet to accomplish, I retired, determining to await the onset of labor.

In two hours I was called, the labor having come on in earnest, and in another hour the labor was completed by the birth of a fine, healthy child. The hemorrhage occurred as usual; and after five hours of as exhausting work as often falls to the lot of the physician, I left her free from danger.

In an early paragraph of this paper I said that no class of cases is nature and the obstetrician more likely to be nonplussed than in these. Nature *is*, however, sometimes competent to effect the delivery, and does so in those cases, denominated by writers "spontaneous podalic version." This case only happens when the child is relatively very small, and is a very rare occurrence indeed. In practice it hardly need be dreamed of as a means likely to extricate the doctor and patient from a position of extreme embarrassment and danger. The doctor needs all his resources and the patient all the courage she can muster; and even under the best management these cases must always be a source of trial and trouble.

